# Mitochondrial protective effects caused by the administration of mefenamic acid in sepsis

**DOI:** 10.1186/s12974-022-02616-6

**Published:** 2022-11-04

**Authors:** Diogo Dominguini, Monique Michels, Leticia B. Wessler, Emilio L. Streck, Tatiana Barichello, Felipe Dal-Pizzol

**Affiliations:** 1 Laboratory of Experimental Pathophysiology, Graduate Program in Health Sciences, University of Southern Santa Catarina (UNESC), Criciúma, SC 88806-000 Brazil; 2 Laboratory of Bioenergetics, Graduate Program in Health Sciences, University of Southern Santa Catarina (UNESC), Criciúma, SC 88806-000 Brazil; 3 Translational Psychiatry Program, Department of Psychiatry and Behavioral Sciences, McGovern Medical School, The University of Texas Health Science Center at Houston, Houston, TX 77054 USA

**Keywords:** Neuroinflammation, Sepsis, NLRP3, Mefenamic acid, Mitochondria, TFAM, 8-oxoG, NLRP-3

## Abstract

The pathophysiology of sepsis may involve the activation of the NOD-type receptor containing the pyrin-3 domain (NLPR-3), mitochondrial and oxidative damages. One of the primary essential oxidation products is 8-oxoguanine (8-oxoG), and its accumulation in mitochondrial DNA (mtDNA) induces cell dysfunction and death, leading to the hypothesis that mtDNA integrity is crucial for maintaining neuronal function during sepsis. In sepsis, the modulation of NLRP-3 activation is critical, and mefenamic acid (MFA) is a potent drug that can reduce inflammasome activity, attenuating the acute cerebral inflammatory process. Thus, this study aimed to evaluate the administration of MFA and its implications for the reduction of inflammatory parameters and mitochondrial damage in animals submitted to polymicrobial sepsis. To test our hypothesis, adult male Wistar rats were submitted to the cecal ligation and perforation (CLP) model for sepsis induction and after receiving an injection of MFA (doses of 10, 30, and 50 mg/kg) or sterile saline (1 mL/kg). At 24 h after sepsis induction, the frontal cortex and hippocampus were dissected to analyze the levels of TNF-α, IL-1β, and IL-18; oxidative damage (thiobarbituric acid reactive substances (TBARS), carbonyl, and DCF-DA (oxidative parameters); protein expression (mitochondrial transcription factor A (TFAM), NLRP-3, 8-oxoG; Bax, Bcl-2 and (ionized calcium-binding adaptor molecule 1 (IBA-1)); and the activity of mitochondrial respiratory chain complexes. It was observed that the septic group in both structures studied showed an increase in proinflammatory cytokines mediated by increased activity in NLRP-3, with more significant oxidative damage and higher production of reactive oxygen species (ROS) by mitochondria. Damage to mtDNA it was also observed with an increase in 8-oxoG levels and lower levels of TFAM and NGF-1. In addition, this group had an increase in pro-apoptotic proteins and IBA-1 positive cells. However, MFA at doses of 30 and 50 mg/kg decreased inflammasome activity, reduced levels of cytokines and oxidative damage, increased bioenergetic efficacy and reduced production of ROS and 8-oxoG, and increased levels of TFAM, NGF-1, Bcl-2, reducing microglial activation. As a result, it is suggested that MFA induces protection in the central nervous system early after the onset of sepsis.

**Supplementary Information:**

The online version contains supplementary material available at 10.1186/s12974-022-02616-6.

## Introduction

Sepsis is a significant cause of death in the intensive care unit (ICU) [1], as its development is related to an exacerbated inflammatory response that affects all body systems, including the central nervous system (CNS)[2].

Sepsis development is partially related to immune system dysfunction by activating pattern recognition receptors (Toll-Like (TLR) and NOD-Like receptors). The NOD-type receptor containing the pyrin-3 domain (NLRP3) is found in the intracellular environment, mainly in myeloid lineage cells such as microglia [3]. In addition, it is related to several brain pathologies (including Alzheimer's disease, Parkinson's disease, amyotrophic lateral sclerosis, traumatic brain injury). NLRP3 activation can induce neuroinflammation, production of reactive oxygen species (ROS), and damage to cellular components [4–6].

First, TLR needs to be activated, leading to the translocation of the nuclear transcription factor Кappa B (NF-κB) to the cell nucleus, promoting the transcription and translation of the NLRP3 protein [7]. For assembly of the inflammasome, a second stimulus is needed, which can be generated by the formation of pores, promoting the flow of ions (K^+^, Ca^2+^_,_ and H^+^) or by lysosomal destabilization, or even by damage-associated molecular patterns (DAMPs) [8]. When stimulated, the C-terminal caspase adapter protein (ASC) is recruited, following the NLRP3 oligomerization process and the cleavage of pro-caspase 1. With the activation of this complex and caspase-1 activation, maturation occurs releasing IL-1β and IL-18, generating a rapid and intense inflammatory response, promoting cell death [3, 9].

Mitochondrial dysfunctions in sepsis occur by several mechanisms, leading to oxidative damage (including mitochondrial DNA (mtDNA) and stimulating NLRP3 activity [10]. One of the products of mtDNA oxidation is 8-oxoguanine (8-oxoG) [11]. 8-oxoG accumulation in mtDNA is known to induce cognitive dysfunction, amyloid beta protein (Aβ) deposition, and neuronal death, suggesting that mtDNA protection against oxidative stress is crucial for the maintenance of neuronal function [11]. Several studies highlight the role of NLRP3 in CNS disorders; however, the use of inhibitors can attenuate this response, preventing brain damage and worsening of neuroinflammation, improving possible neurological dysfunction [12–14].

NLRP3 inhibitors such as mefenamic acid (MFA) can decrease the activity of inflammasome and attenuate inflammatory responses and may be a therapeutic target in inflammatory disorders [15–18]. Therefore, this work aims to evaluate the administration of MFA and its implications in the reduction of inflammatory parameters, mitochondrial and cognitive damage in animals submitted to sepsis.

## Material and methods

### Animals

Male Wistar rats (60 days old, weighing 200 to 300 g) were obtained from the Universidade do Extremo Sul Catarinense (UNESC) breeding colony. The animals were housed in groups of five with water and food available ad libitum and were maintained on a 12-h light–dark cycle (lights on at 7:00 am), at a temperature of 23 ± 1 °C. These conditions were maintained constant throughout the experiments. All experimental procedures were performed with the approval of the local Ethics Committee of Animals Use (Protocol 029/2017-2).

### Experimental procedure

Wistar rats were submitted to sepsis by the CLP [19] model and divided into four experimental groups. Immediately after sepsis induction, the animals received an intraperitoneal injection of MFA at doses of 10, 30, and 50 mg/kg or saline. At 24 h after sepsis induction, the animals were euthanized, and the cerebral structures (frontal cortex and hippocampus) were dissected for further analysis (see below), with *n* = 6 animals for each group. A separate cohort of animals were followed for 15 days and the survival rate was analyzed.

### Sepsis induction

Rats were subjected to cecal ligation and perforation (CLP), as previously described by Fink and Heard (1990) [20]. Briefly, animals were anesthetized using a mixture of ketamine (80 mg/kg) and xylazine (10 mg/kg), given intraperitoneally. Under aseptic conditions, a 3-cm midline laparotomy was performed to allow exposure of the cecum with the adjoining intestine. The cecum was tightly ligated with a 3.0-silk suture at its base, below the ileocecal valve, and perforated once with a 14-gauge needle. The cecum was then gently squeezed to extrude a small amount of feces from the perforation site, returned to the peritoneal cavity, followed by the closure of the laparotomy with 4.0-silk sutures. The rats were resuscitated with normal saline (50 mL/kg subcutaneously) immediately and 12 h after CLP. All animals were returned to their cages with free access to water and food. The rats were submitted to all surgical procedures in the sham-operated group, but the cecum was neither ligated nor perforated. Rats were divided in 5 experimental groups: (1) Sham + saline, (2) CLP + saline, (3) CLP + MFA 10 mg/kg, (4) CLP + MFA 30 mg/kg, and (5) CLP + MFA 50 mg/kg.

### Dissection of brain structures

After 24 h, rats were euthanatized by decapitation and brain transferred within 1 min to ice-cold isolation buffer (0.23 M mannitol, 0.07 M sucrose, 10 mM Tris–HCl, and 1 mM EDTA, pH 7.4). The prefrontal cortex and hippocampus were dissected in the ice-cold buffer in a Petri dish, and after brain structures were rapidly frozen and stored at 80 ºC.

### Cytokines levels

Brain samples were homogenized in extraction solution containing PBS buffer pH 7.0, centrifuged at 10,000×*g* for 3 min, and 100 μL of the supernatant as used for each assay. The concentration of cytokines TNF-α (*DY510, R&D System*), IL-1β *(DY501, R&D System),* IL-6 (*DY506, R&D System*), and IL-18 *(EPR22249-263, Abcam)* was determined by enzyme-linked immunosorbent assay (ELISA) on a microplate reader using a commercially available kits *(R&D System)*. Six animals for each group were used.

### Thiobarbituric acid reactive species levels

Oxidative damage in lipids was assessed by the formation of TBARS during an acid-heating reaction, as previously described by Draper and Hadley et al., (1990) [21]. The samples were mixed with 1 ml of trichloroacetic acid 10% and 1 ml of thiobarbituric acid 0.67% and then heated for 30 min. TBARS levels were determined spectrophotometrically by the absorbance at 532 nm.

### Protein carbonyls levels

Oxidative damage to proteins was measured through the determination of carbonyl groups content based on the reaction with dinitrophenylhydrazine (DNPH), as previously described by Levine et al. [22]. Proteins were precipitated by adding 20% trichloroacetic acid and were redissolved in DNPH. The absorbance was monitored spectrophotometrically at 370 nm.

### Protein determination

Oxidative damage parameters and cytokines levels were normalized to the protein content according to Lowry et al., (1951) [22, 23].

### Immunohistochemistry

Another batch of animals (five per group) was used to perform immunohistochemistry. Five animals per group were perfused with 0.9% sterile saline for 10 min (flow rate 20 mL/min) followed by 10 min with paraformaldehyde (PFA) solution 4% in PBS (pH 7.4) (flow rate 20 mL/min). After, 5-μm sections from the hippocampus and prefrontal cortex were incubated in 0.5% hydrogen peroxide in 0.1 M PBS (pH 7.4) for 30 min at room temperature to block endogenous peroxidase activity. After washing with PBST, sections were incubated for 30 min with PBST containing 2% bovine serum albumin to block non-specific protein binding. Sections were then incubated overnight at 4 °C with a rabbit monoclonal IgG antibody against Iba1 (Abcam—ab178846) (1:400 dilution). After washing with PBST, sections were incubated at room temperature for 1 h with biotinylated anti-rabbit IgG (1:100 dilution; Abcam). Sections were incubated with 3,3′-diaminobenzidine (DAB) (Spring Bioscience). Sixteen random images per brain section were acquired at × 200 magnification. The positive control was used in all groups, according to the datasheet of the antibody. The increase × 200 provides a more detailed expansion of the CA3 region and dentate gyrus and prefrontal cortex. The slices were then visualized using an inverted microscope (Eclipse Ti-U-Nikon Instruments) coupled to a digital camera. Manual selection of stained tissue was performed, and this was quantified using the “Threshold Color” plugin for the ImageJ software [24].

### Assessment of the activity of mitochondrial complexes

To evaluate mitochonrdrial electron transport chain alterations complex activities were evaluated. Complex I activity was determined according to Cassina and Radi (1996)[25] by reading the NADH-dependent rate of ferricyanide reduction at 420 nm. Complex II activity was evaluated by the method described by Fischer et al. (1985)[26], where the incubation medium consisted of potassium phosphate, sodium succinate and 2,6-dichloroindophenol (DCIP). Initially, the samples were pre-incubated with 40–80 mg of proteins from the homogenate, at 30 °C, for 20 min. Then, 4 mM sodium azide and 7 mM rotenone were added to the medium, and the reaction started with the addition of 40 M DCIP. Absorbances were recorded for 5 min at 600 nm.

The activity of complex II–III was determined according to Fischer et al. (1985)[26], in which the reaction medium, consisting of potassium phosphate (40 mM, pH 7.4), containing sodium succinate (16 mM), was pre-incubated with 40–80 mg of homogenate proteins at 30 °C for 30 min. Then, 4 mM sodium azide and 7 mM rotenone, and addition of 0.6 mg/ml of cytochrome c. Absorbances were recorded for 5 min at 550 nm.

The activity of complex IV (cytochrome c oxidase) was determined according to Rustin et al. (1994)[27] where the incubation medium was composed of potassium phosphate buffer (10 mM, pH 7.0), dodecyl-maltoside (0.6). mM) and 10–20 mg protein (homogenized). The reaction was started with the addition of 0.7 mg of reduced cytochrome c. Cytochrome c oxidase activity was measured by the decrease in absorbance due to the oxidation of previously reduced cytochrome c. Readings were taken at 550 nm.

### Western blotting

The NRLP3, Bax, and Bcl-2 protein levels were determined by Western blotting in samples from the hippocampus and prefrontal cortex. Briefly, samples were homogenized in Laemmli-sample buffer (62.5 mM Tris–HCl, pH 6.8, 1% (w/v) SDS, and 10% (v/v) glycerol) and equal amounts of protein (30 μg/well) were fractionated by SDS-PAGE and electro-blotted onto nitrocellulose membranes. Protein loading and electro-blotting efficiency were verified through Ponceau S staining, and the membrane was blocked in Tween–Tris-buffered saline (TTBS; 100 mM Tris–HCl, pH 7.5, containing 0.9% NaCl and 0.1% Tween-20) containing 5% albumin. Membranes were incubated overnight at 4 °C with primary antibody diluted at 1:1000 in TTBS (NRLP3 (Abcam—ab214185), Bax (Abcam—ab32503) and Bcl-2 (Abcam—ab194583)) and washed with TTBS. Anti-IgG from mouse or rabbit (according to the species that originated the primary antibody) linked to peroxidase was incubated with the membrane for an additional 2 h at room temperature (1:5000 dilution range), the membrane was washed, and the immunoreactivity was detected by enhanced chemiluminescence using ECL Chemiluminescence kit. Densitometric analysis of the films was performed with ImageJ software. Blots were developed to be linear in the range used for densitometry.

### Mitochondrial isolation

Mitochondrial isolation was performed according to Rosenthal et al. (1987). Brain samples were homogenized in a buffer containing 225 mM mannitol, 75 mM sucrose, 1 mM EGTA, 0.1% bovine albumin, 10 mM HEPES, pH 7.2. The homogenate was centrifuged at 2000 × g, for 3 min, at 4 °C. After this centrifugation, the supernatant was again centrifuged at 12,000 × *g*, for 8 min, at 4 ºC. The pellet was resuspended in 12 mL of isolation buffer containing 20 µL of 10% digitonin and centrifuged at 12,000 × g for 10 min at 4 °C. The sediment from this centrifugation was resuspended in isolation buffer without EGTA and centrifuged at 12,000 × *g* for 10 min at 4 °C. The pellet containing purified mitochondria was resuspended in isolation buffer without EGTA at a final protein concentration of approximately 20 mg/ml. After isolation, mitochondria were quickly frozen in a freezer at − 80 °C for further analysis.

### ROS levels in mitochondria

Intramitochondrial ROS production was detected using the non-fluorescent cell permeating compound, 2′7′-dichlorofluorescein diacetate (DCFH-DA). Intracellular esterases hydrolyzed DCFH-DA in dichlorofluorescein (DCFH). This non-fluorescent molecule is then oxidized to fluorescent dichlorofluorescein (DCF) by the action of cellular oxidants. Mitochondrial samples were treated with DCFH-DA (10 µM) for 30 min at 37 °C. After exposure to DCFH-DA, samples were washed with PBS solution with 0.2% Triton X-100. Fluorescence was measured in a plate reader (Spectra Max GEMINI XPS, Molecular Devices, USA) with excitation at 485 nm and emission at 520 nm. Values were expressed as fluorescence units/µg protein.

### Analysis of 8-oxoG levels

The mtDNA was extracted from the tissues of the frontal cortex and hippocampus. The extraction and quantification of mtDNA were performed using commercial kits provided by Abcam. Samples were homogenized with Cytosol Extraction Buffer. Soon after, the Mitochondrial Lysis buffer and ethanol were added. Finally, mtDNA was resuspended and purified for use in the 8-oxoG analysis. For the analysis of the 8-oxoG level, the immunoassay test (ELISA Sandwich) was used through the Cell kit, Biolabs®. The procedures for each dosage were performed according to the manufacturer's recommendations. The reaction was stopped with the addition of 100 μL of stop solution and read in a spectrophotometer, using a wavelength of 450 nm.

### RT-PCR analysis

Total RNA was isolated with Trizol® reagent (Invitrogen, USA) following the manufacturer’s instructions. To eliminate genomic DNA contamination, the total RNA was quantified by spectrophotometry (A260/280 nm) and then treated with Deoxyribonuclease I (Invitrogen). Following the manufacturer's instruction, the cDNA was synthesized with ImProm-II™ Reverse Transcription System. Quantitative PCR was performed using SYBR®Green (Invitrogen) in a 7500 Fast Real-Time System Software v.2.0.5 (Applied Biosystems). Primers (TFAM, e NRF1) are in Additional file 1: Figure S1). Previous results from our laboratory demonstrated that the expression of these genes was different when compared with Sham and CLP animals [28]. Thus, based on the 3Rs principle, a sham group was not included in gene analysis, and all comparisons were performed between CLP-treated and not-treated animals.

### Statistical analysis

The Statistical Package for the Social Sciences (SPSS) 20.0 and GraphPad Prism version 7.0 was utilized for statistical analyses. The immunohistochemistry analysis was done through DAB staining for each marker and quantified by the ImageJ program. Data from oxidative brain damage, cytokines, and immunohistochemistry were analyzed by factorial ANOVA (one-factor depending—post hoc Tukey) and expressed as mean ± SEM. Standard Kaplan–Meier mortality curves were compared by the log-rank test. For all comparisons, *p* < 0.05 was considered statistically significant.

## Result

**Fig. 1:**
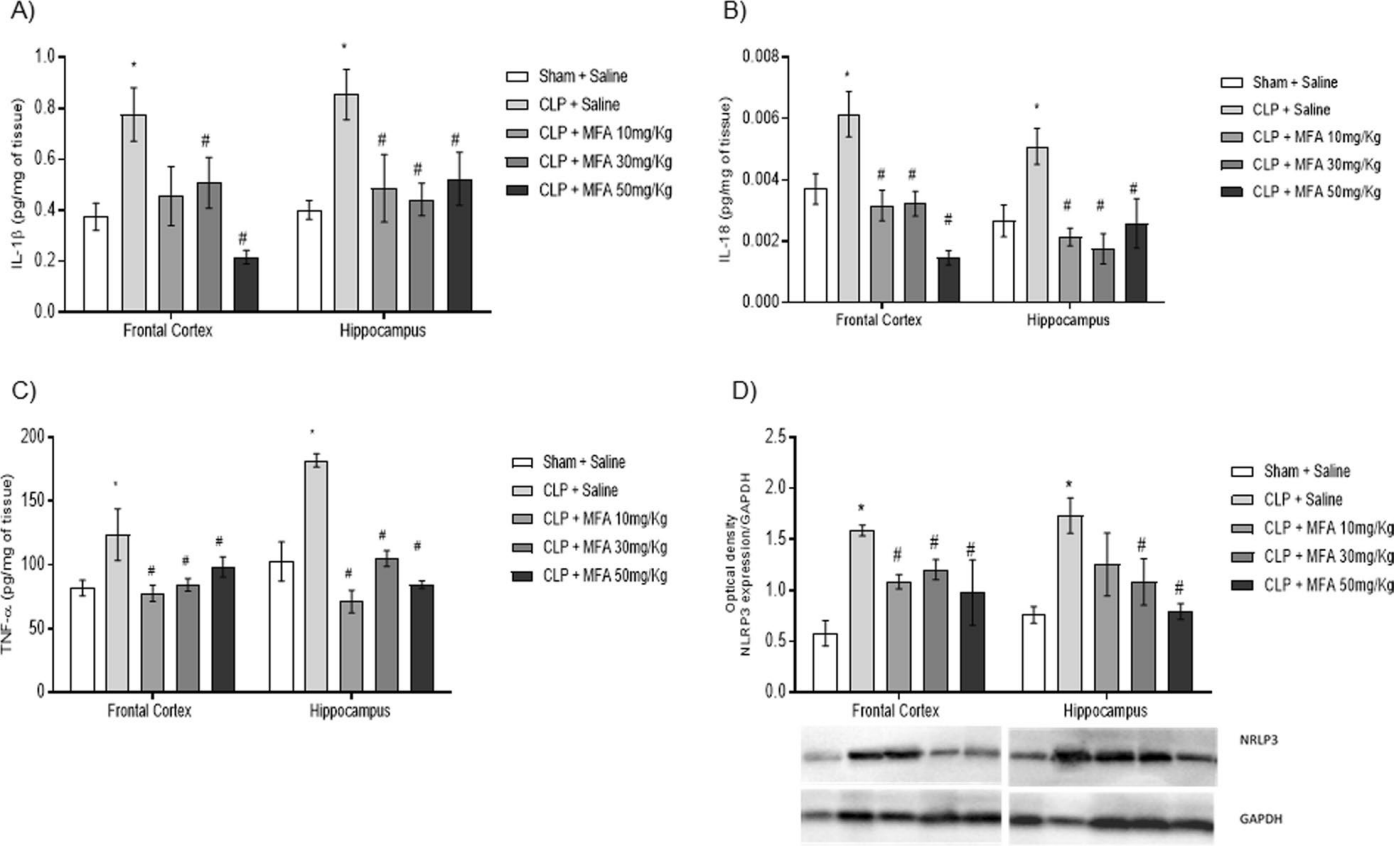
Mefenamic acid decreases brain cytokines and NLRP3 levels and microglial activation in the brain of septic animals. Sepsis was induced by cecal ligation and perforation and mefenamic acid (10, 30 and 50 mg/kg) was administered immediately after sepsis induction. Twenty-four hours after rats were euthanized and the prefrontal cortex and hippocampus were isolated to the determination of **A** IL-1β, **B** IL-18, **C** TNF-α and **D** NLRP3 levels. Data are presented as mean ± SEM, with the statistical difference calculated using the one-way ANOVA test followed by Tukey's post hoc test. *p < 0.05 vs Sham + saline and ^#^*p* < 0.05 vs CLP + saline. *n* = 8 per group

**Fig. 2:**
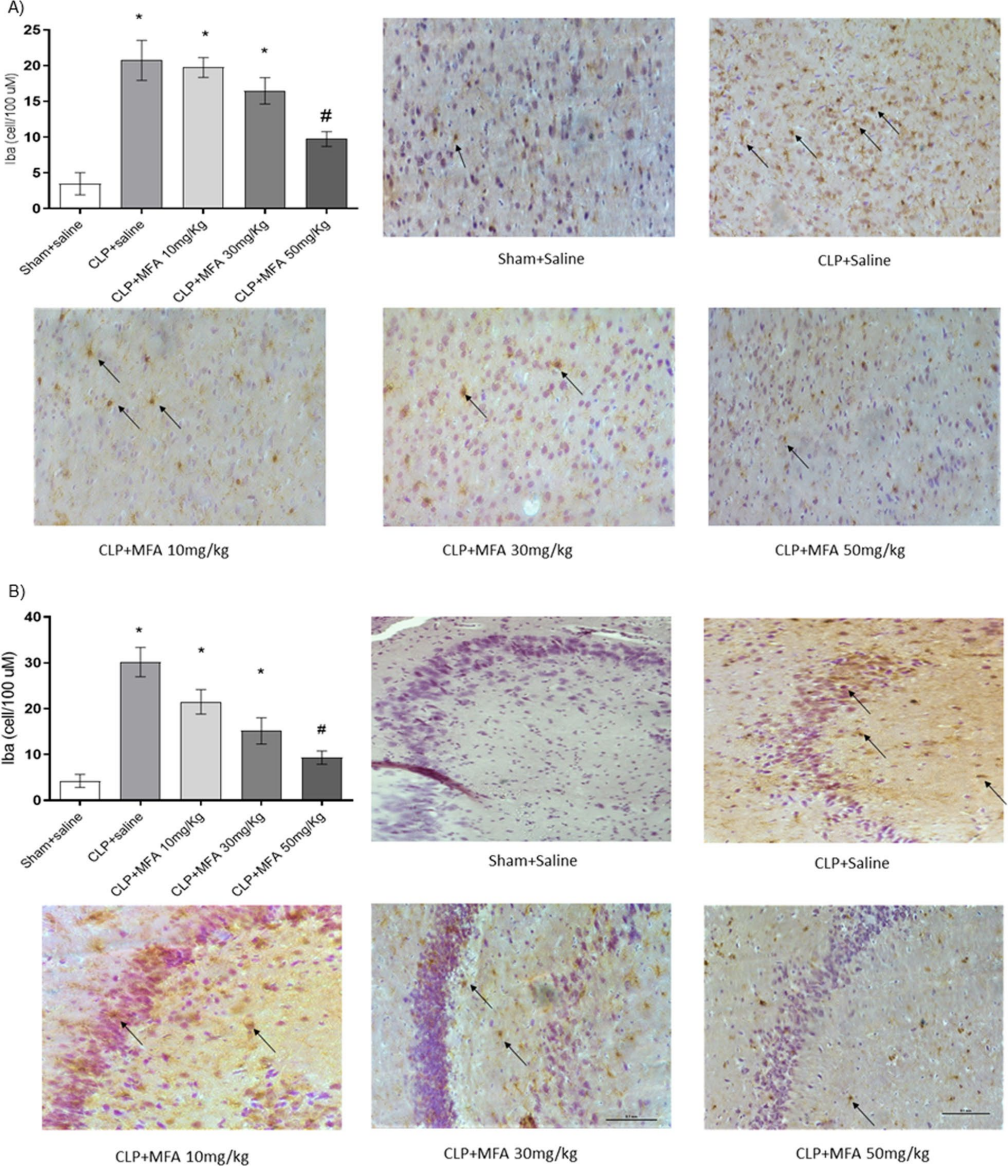
Mefenamic acid decreases microglial activation in the brain of septic animals. Sepsis was induced by cecal ligation and perforation and mefenamic acid (10, 30 and 50 mg/kg) was administered immediately after sepsis induction. Twenty-four hours after rats were euthanized and the prefrontal cortex (**A)** and hippocampus (**B)** were isolated to the determination IBA-1 positive cells. Data are presented as mean ± SEM, with the statistical difference calculated using the one-way ANOVA test followed by Tukey's post hoc test. *p < 0.05 vs Sham + saline and ^#^*p* < 0.05 vs CLP + saline. *n* = 8 per group

Inflammation is widely established as related to the pathophysiology of sepsis, thus first it was determined the effect of MFA on brain cytokines levels (Fig. 1). Cytokine levels were increased at all analyzed structures, and MFA was able to decrease all cytokines to sham levels (Fig. 1A–C). Since it is well known that MFA could decrease NRLP3 activation that is highly expressed in the microglia, it was also measured NRLP3 levels (Fig. 1D), and microglial activation (Fig. 2A, B). There was a significantly increase in NRPL3 levels and IBA-1 positive cells after sepsis, and this was attenuated by MFA treatment.

**Fig. 3:**
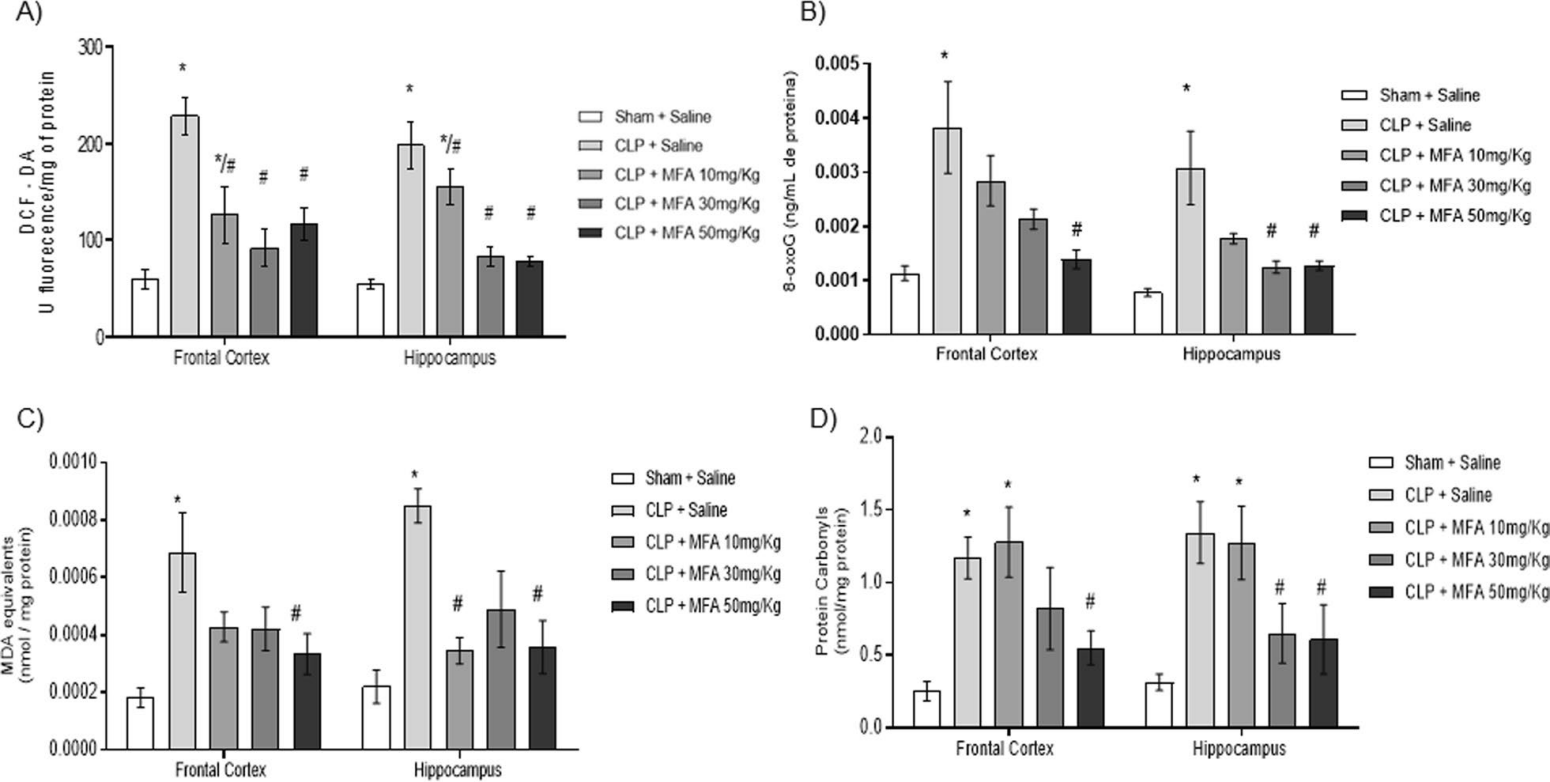
Mefenamic acid decreases brain mitochondrial and mitochondrial reactive oxygen species and oxidative damage in septic animals. Sepsis was induced by cecal ligation and perforation and mefenamic acid (10, 30 and 50 mg/kg) was administered immediately after sepsis induction. Twenty-four hours after rats were euthanized and the prefrontal cortex and hippocampus were isolated to the determination of **A** DCF, **B** 8-oxo guanosine, **C** MDA and **D** carbonyls levels. Data are presented as mean ± SEM, with the statistical difference calculated using the one-way ANOVA test followed by Tukey's post hoc test. *p < 0.05 vs Sham + saline and ^#^*p* < 0.05 vs CLP + saline. *n* = 8 per group

One of the effector signals for triggering the activation of NLRP3 is the production of ROS that induces inflammasome holomerization. The mitochondrial production of ROS was increased in both studied structures after sepsis (Fig. 3A), and this was attenuated by MFA. Both inflammation and mitochondrial ROS production could induce oxidative damage that participates in organ dysfunction that followed sepsis. It is demonstrated in Fig. 3B–D that both mitochondrial (measured by 8-oxo-G) and tissue (measured by TBARS and carbonyl assays) oxidative damage occurred in the prefrontal and hippocampus of septic animals. MFA treatment attenuated both mitochondrial and tissular oxidative damage (Fig. 3B–D), and this effect was more evident to the 30 and 50 mg/kg dose.

**Fig. 4:**
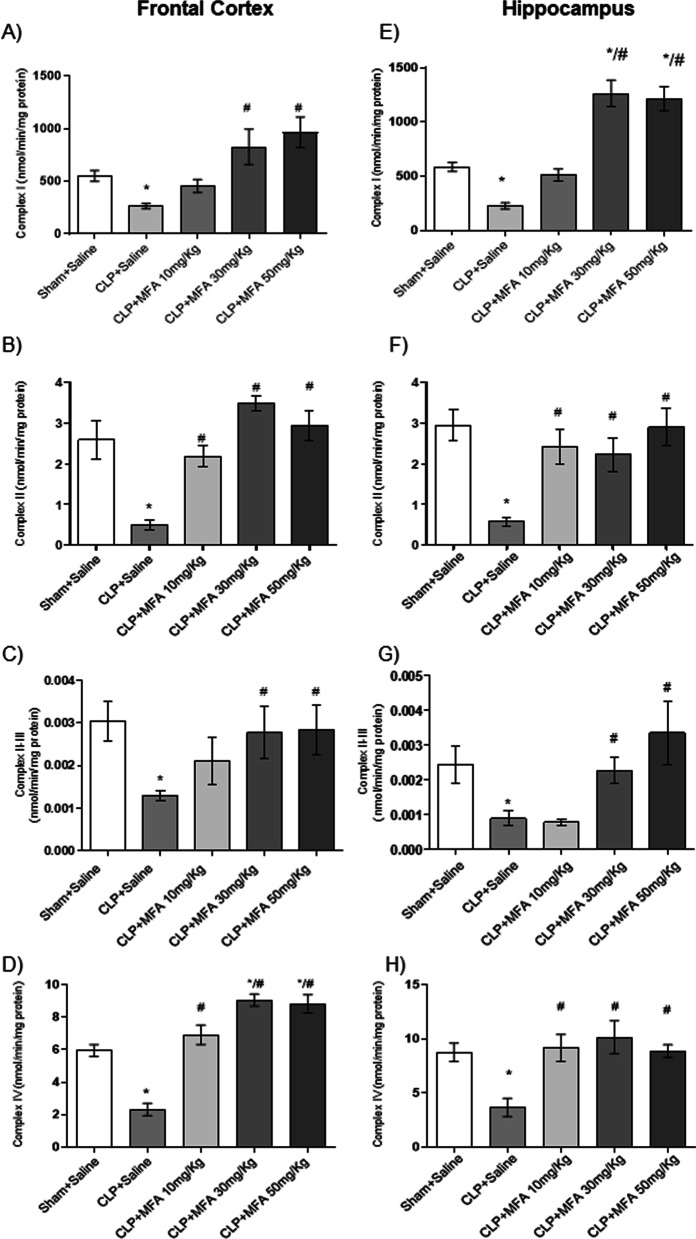
Mefenamic acid improves the activity of mitochondrial respiratory chain complexes in the brain of septic animals. Sepsis was induced by cecal ligation and perforation and mefenamic acid (10, 30 and 50 mg/kg) was administered immediately after sepsis induction. Twenty-four hours after rats were euthanized and the prefrontal cortex and hippocampus were isolated to the determination of the activity of mitochondrial **A**–**E** complex I, **B**–**F** complex II, **C**–**G** complex II–III and **D**–**H** complex IV in frontal cortex (**A**–**D)** and hippocampus (**E–H**). Data are presented as mean ± SEM, with the statistical difference calculated using the one-way ANOVA test followed by Tukey's post hoc test. *p < 0.05 vs Sham + saline and ^#^*p* < 0.05 vs CLP + saline. *n* = 5 per group

**Fig. 5:**
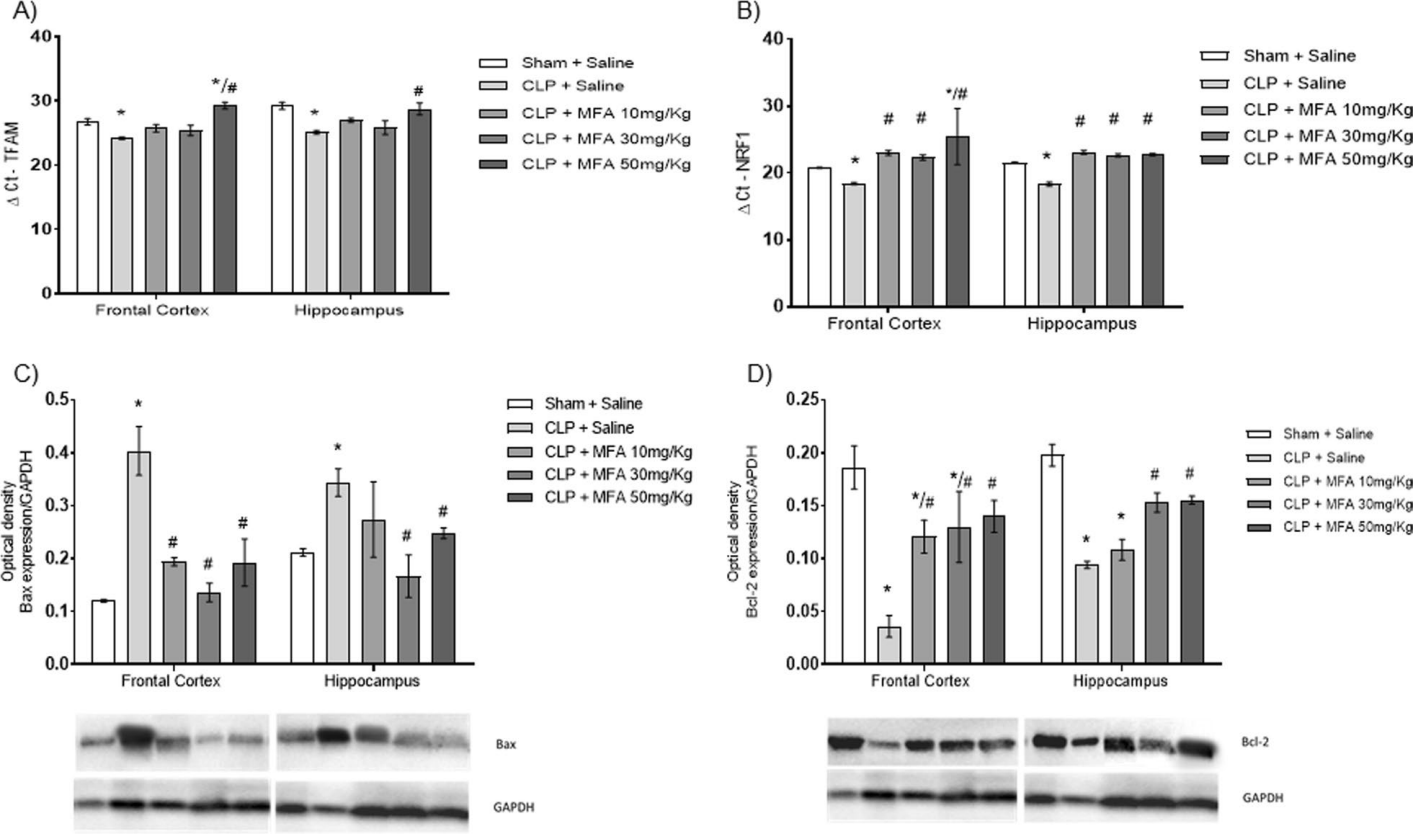
Mefenamic acid reverses the increase in TFAM and NRF1 gene expression and regulates Bax and Bcl-2 protein content in the brain of septic animals. Sepsis was induced by cecal ligation and perforation and mefenamic acid (10, 30 and 50 mg/kg) was administered immediately after sepsis induction. Twenty-four hours after rats were euthanized and the prefrontal cortex and hippocampus were isolated to the determination of the gene expression of **A** TFAM and **B** NRF-1. It was also measured protein content of **C** the pro-apoptotic Bax protein and **D** the anti-apoptotic Bcl-2 protein. Gene expression was quantified by the ∆Ct. Data are presented as mean ± SEM, with the statistical difference calculated using the one-way ANOVA test followed by Tukey's post hoc test. *p < 0.05 vs Sham + saline and ^#^*p* < 0.05 vs CLP + saline. *n* = 5 per group

One of the main effects of brain oxidative damage and inflammation is mitochondrial electron chain dysfunction. It was observed that in the frontal cortex and in the hippocampus of the animals in the CLP + Saline group, there was a decrease in the mitochondrial electron transport activity of complexes I, II, II–III, and IV (Fig. 4) when compared to the Sham + saline. It is noteworthy that septic animals treated with MFA mainly at doses of 30 and 50 mg/kg, in both structures, showed a significant increase in the activity of complexes I, II, II–III, and IV compared to the CLP group + saline. As a response to the septic insult it was found a small, but significant increase in TFAM and NRF-1 gene expression, that was reversed by MFA (Fig. 5A, B), reinforcing its protective effects upon mitochondria. We further evaluated the protein content of the proteins of the BCL-2 family. Sepsis induced a significant increase in Bax and a decrease in Bcl-2 levels (Fig. 5C, D). However, animals treated with MFA had a lower concentration of the pro-apoptotic Bax protein and a higher concentration of the anti-apoptotic Bcl-2 protein (Fig. 5C, D). This may explain the effect upon mitochondrial dysfunction and consequently a possible decrease in the interaction between mitochondria and NLRP3 activation.

All these protective effects upon the brain are reflected in the periphery. MFA was able to slightly, but significantly, improve mortality in the higher used doses (Additional file 1: Fig. S1 and Additional file 2: Fig. S2) and plasma cytokines (Additional file 3: Fig. S3).

## Discussion

The present study aimed to evaluate the modulation of NLRP3 activity by administering MFA and its implications in reducing inflammatory parameters, mitochondrial and oxidative damage in the early stages of sepsis. The 24-h proinflammatory cytokine levels were higher, and these data corroborate with previous results, which indicate increased concentrations of IL-1β and TNF-α early after sepsis, with a peak increase between 18 and 48 h after CLP induction [29–31]. This increase in cytokines in the CNS maybe related to the breakdown of the BBB, increasing the passage of inflammatory mediators into the brain tissue, stimulating microglial cells [30–33]. In another study, high concentrations of IL-1β and TNF-α and a large number of cells labeled with IBA-1 were found in the early stages of sepsis in an animal model of sepsis induced by the CLP surgery [34, 35]. It is known that the increase in IL-18 is related to septic shock and tissue damage, and its control is critical [36]. It is noteworthy that the production of these cytokines may persist at later times, regulating inflammatory pathways, angiogenesis, and cognition, and may contribute to prolonging or worsening brain dysfunction [24].

MFA, especially at the highest dose, reduced cytokine levels (IL-1β, IL-18, and TNF-α) in the early stages of sepsis, favoring a balance of the immune system and of equilibrium of inflammatory components, crucial for the development of a favorable outcome. However, a recently discovered mechanism of action shows that this drug is an effective and selective inhibitor of the NLRP3 inflammasome through inhibition of the volume-regulated anion channel [18, 37–39]. It is proposed that if there is no oligomerization of the inflammasome, there would be no cleavage and maturation of IL-1β and IL-18, which is a possible justification for the decrease in these cytokines observed in the present study. The reduction in these cytokines caused by the administration of MFA was related to the lower expression of NLRP3 in brain tissues [24, 40–43].

In fact, in the present study, MFA inhibited the NLRP3 axis and acted by decreasing IL-1β and IL-18 in microglia, followed by modulation of neuroinflammation and reduction in neuronal oxidative damage [18, 37]. Oxidative damage to lipids and proteins in the early stages of sepsis is a well-known phenomenon. Still, the increased activity of NLRP3 can trigger ROS production, which is a mechanism of damage to cellular components, and may activate pathways involved in neuronal death [5, 34, 35, 44, 45].

In the literature, there are still little data on the relationship between MFA and NLRP3 [46, 47]. Studies in neuron cultures showed that doses of 5 μM of MFA are enough to reduce oxidative stress [16, 48]. In an animal model of excitotoxicity, treatment with MFA at a dose of 25 mg/kg provided significant protection against increased lipid peroxidation, protein oxidation, TNF-α, IL-1β, and Bax levels, thus decreasing neurotoxicity in rats subjected to an inflammatory process, corroborating the findings of this study at doses of 30 and 50 mg/kg [48].

In sepsis, a mitochondrial dysfunction is an important event in NLRP3 inflammasome activation, as NLRP3 stimuli activate the mitochondrial OxPhos machinery to increase ROS and decrease mitochondrial membrane potential (mtΔΨ) and respiratory chain complex activity [5, 49]. Complexes I, III, and IV play a fundamental role in regulating ROS production. When there are changes in their activities, they can generate a stimulus (higher OxPhos proteins), causing NLRP3 oligomerization, increasing the inflammatory process [50]. MFA increased the activity of mitochondrial complexes, especially complexes I, II, and IV, at doses of 30 and 50 mg/kg, in both structures evaluated.

MFA may be related to a better oxidative bioenergetic efficiency of the organism and stability in the electrical potential. It may be a stimulus for mitochondrial biogenesis, attenuating the rate of structural protein synthesis, duplication of organelles, and the increase in the speed of nerve conduction and synaptic transmission, decreasing the impact of sepsis on brain tissue [51–53]. Treatment with MFA protected against mitochondrial damage, causing a reduction in ROS production and preserving the content of Mn-SOD, corroborating the findings of Armagan et al., (2012)[48] in an animal model d-serine inflammation and Kim et al., (2017)[54], in neuronal culture.

Assuming that in an inflammatory process, the mechanism involved with inflammasome activation is the binding of mtDNA directly to NLRP3; this association depends on the oxidation by 8-oxoG [55]. Thus, results from the present study indicate, for the first time, that the increase in NLRP3 inflammasome activity in the septic group was related to the increase in 8-oxoG in mtDNA, in both studied structures. In addition, with the evolution of sepsis, there is a direction to the activation pathway of the NLRP3 inflammasome, becoming a cycle, culminating in the damage of the neuronal tissue generated by the deposit of 8-oxoG [43, 56–58]. Depending on the amount of 8-oxoG, the greater are the changes in the bioenergetics of cells and tissues, which may evolve to the loss of their function [59].

The present study is pioneering, showing the action of MFA in decreasing 8-oxoG in the cerebral tissues of the frontal cortex and hippocampus in CLP animals 24 h after sepsis. It is noteworthy that the lower levels of 8-oxoG, generate a state of mtDNA protection, which according to a study by Oka et al. [11], is related to the increase in TFAM. TFAM stimulation can improve redox and mitochondrial activity, motor function, and high survival rates in a sepsis model [60, 61]. The literature shows evidence that septic shock and organ failure evolve to mortality and may be related to mitochondrial dysfunction and lack of bioenergetic recovery [42, 43, 62].

Mitochondrial recovery depends on the upregulation of mitochondrial biogenesis [63, 64]. Notably, the administration of MFA amplifies the expression and activation of factors that stimulate biogenesis and mitochondrial repairs, such as TFAM and NRF-1. Mitochondrial biogenesis is related to proteins of the Bcl [31, 65, 66]. MFA increased the expression of Bcl-2, which can decrease cellular apoptosis through balance with the Bax protein and the production of antioxidants [5, 65]. Furthermore, a previous study demonstrated that MFA in cerebral ischemia and reperfusion model increased the expression of anti-apoptotic proteins such as Bcl-xl and Bcl-2 and decreased Bax concentrations, improving energy metabolism and response to the inflammatory process, which may be related to the decrease in microglia activity, such as the decrease in Iba-1 [17, 34].

Some limitations of the presented results should be noted. First, MFA is not a specific NRLP-3 inhibitor, some other confounder mechanisms could partially explain its protective effects. Second, a single dose administered immediately after sepsis induction was used. Despite being highly mechanistic relevant, this is of less clinical relevance, thus, later administration and multiple doses regime should be addressed in future studies. Third, it is not possible to precisely determine in our experiments if NLRP3 activation is a cause or consequence of mitochondrial dysfunction, but our results give insights o the relation between these phenomena.

In conclusion, in sepsis, there is an increase in proinflammatory cytokines in brain tissues at early stages, culminating in oxidative damage and NLRP3 activation, which is related to bioenergetic changes, causing the deposition of 8-oxoG in mtDNA. However, MFA performed well in decreasing the activity of NLRP3 and proinflammatory cytokines in the brains of septic rats with consequent impact on survival of animals. The dose of 50 mg/kg seemed to be the most efficient, and it inhibited inflammasome activation, decreasing the IL-1β and IL-18 maturation, decreasing the oxidative damage and the formation of ROS. It is noteworthy that MFA controlled the deposition of 8-oxoG, increasing mitochondrial activity, reducing ROS, increasing mtDNA protection molecules such as TFAM, which could become a future therapeutic target.

## Supplementary Information

**Additional file 1: Figure S1.** List of used primes

**Additional file 2: Figure S2.** Mefenamic acid improves mortality in an animal model of severe sepsis. Sepsis was induced by cecal ligation and perforation and mefenamic acid (10, 30 and 50 mg/kg) was administered immediately after sepsis induction. Animals were followed for 14 days. Standard Kaplan–Meier mortality curves were compared by the log-rank test. *p < 0.05 vs. Sham + saline and ^#^*p* < 0.05 vs. CLP + saline.

**Additional file 3: Figure S3.** Mefenamic acid decreases plasma cytokines levels in septic animals. Sepsis was induced by cecal ligation and perforation and mefenamic acid (10, 30 and 50 mg/kg) was administered immediately after sepsis induction. Twenty-four hours after rats were euthanized and the blood was drawn to the of **A** IL-1β, **B** IL-18 and **C** TNF-α plasma levels. Data are presented as mean ± SEM, with the statistical difference calculated using the one-way ANOVA test followed by Tukey's post hoc test. *p < 0.05 vs Sham + saline and ^#^*p* < 0.05 vs CLP + saline. *n* = 8 per group.

## Data Availability

All data generated or analyzed during this study are included in this published article (and its additional files).

## Abbreviations

8-oxoG: 8-Oxoguanine guanosine
ASC: C-terminal caspase adapter protein
Aβ: Amyloid beta protein
CLP: Cecal ligation and perforation
CNS: Central nervous system
DAMPs: Damage-associated molecular patterns
DCFH-DA: 2′7'-Dichlorofluorescein diacetate
DCIP: 2,6-Dichloroindophenol
IBA-1: Ionized calcium-binding adaptor molecule 1
ICU: Intensive care unit
MFA: Mefenamic acid
mtDNA: Mitochondrial deoxyribonucleic acid
NF-κB: Transcription factor Кappa B
NLPR-3: NOD-type receptor containing the pyrin-3 domain
PFA: Paraformaldehyde
ROS: Reactive oxygen species
TBARS: Thiobarbituric acid reactive substances
TFAM: Mitochondrial transcription factor A
TLR: Toll-like receptor
