# Supplementary figures and images for: Mitochondrial protective effects caused by the administration of mefenamic acid in sepsis
Source: J Neuroinflammation. 2022 Nov 4;19:268. doi: 10.1186/s12974-022-02616-6 (PMC9636698; doi:10.1186/s12974-022-02616-6)

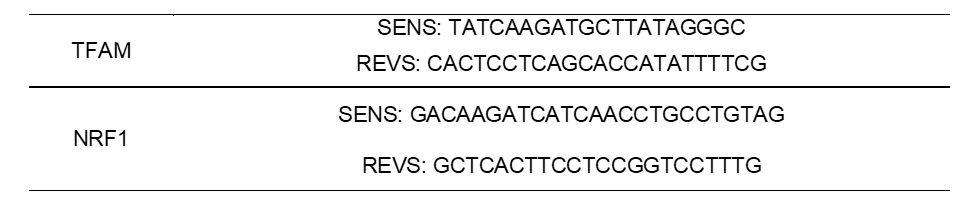

Supplement: Supplementary file 1 — Additional file 1: Figure S1. List of used primes [file 12974_2022_2616_MOESM1_ESM.tif]

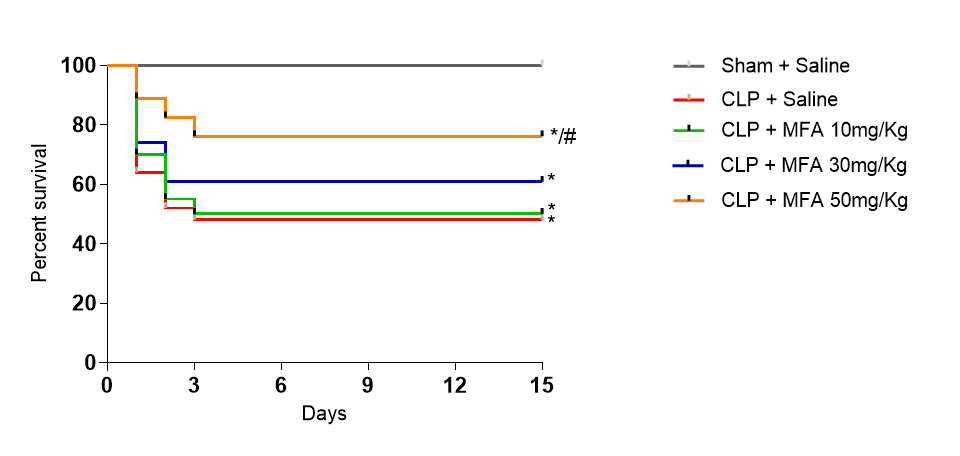

Supplement: Supplementary file 2 — Additional file 2: Figure S2. Mefenamic acid improves mortality in an animal model of severe sepsis. Sepsis was induced by cecal ligation and perforation and mefenamic acid (10, 30 and 50 mg/kg) was administered immediately after sepsis induction. Animals were followed for 14 days. Standard Kaplan–Meier mortality curves were compared by the log-rank test. *p < 0.05 vs. Sham + saline and #p < 0.05 vs. CLP + saline. [file 12974_2022_2616_MOESM2_ESM.tif]

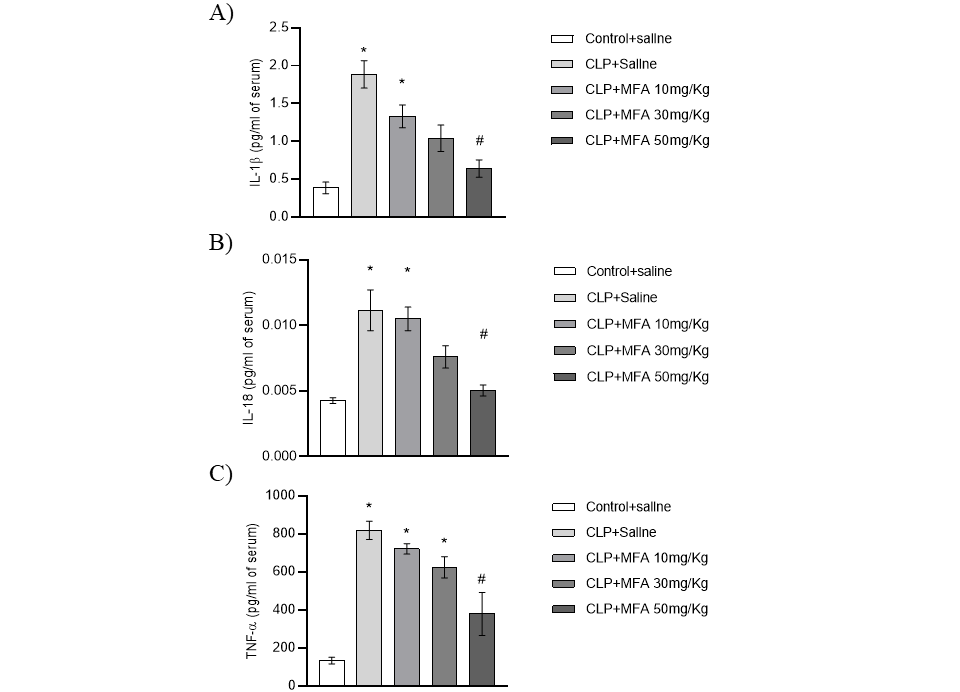

Supplement: Supplementary file 3 — Additional file 3: Figure S3. Mefenamic acid decreases plasma cytokines levels in septic animals. Sepsis was induced by cecal ligation and perforation and mefenamic acid (10, 30 and 50 mg/kg) was administered immediately after sepsis induction. Twenty-four hours after rats were euthanized and the blood was drawn to the of A IL-1β, B IL-18 and C TNF-α plasma levels. Data are presented as mean ± SEM, with the statistical difference calculated using the one-way ANOVA test followed by Tukey's post hoc test. *p < 0.05 vs Sham + saline and #p < 0.05 vs CLP + saline. n = 8 per group. [file 12974_2022_2616_MOESM3_ESM.tif]
